# Loss of Stag2 cooperates with EWS-FLI1 to transform murine Mesenchymal stem cells

**DOI:** 10.1186/s12885-019-6465-8

**Published:** 2020-01-02

**Authors:** Marc El Beaino, Jiayong Liu, Amanda R. Wasylishen, Rasoul Pourebrahim, Agata Migut, Bryan J. Bessellieu, Ke Huang, Patrick P. Lin

**Affiliations:** 1 Department of Orthopaedic Oncology – Unit 1448, MD Anderson Cancer Center, 1515 Holcombe Boulevard, Houston, TX 77030 USA; 2 Department of Bone and Soft Tissue Tumor, Peking University Cancer Hospital & Institute, 52 Fu-Cheng Road, Hai-Dian District, Beijing, 100142 China; 3 Department of Genetics – Unit 1010, MD Anderson Cancer Center, 1515 Holcombe Boulevard, Houston, TX 77030 USA; 4 Department of Leukemia – Unit 428, MD Anderson Cancer Center, 1515 Holcombe Boulevard, Houston, TX 77030 USA

**Keywords:** Ewing sarcoma, Mouse model, Mesenchymal stem cells, p53, EWS-FLI1, Stag2

## Abstract

**Background:**

Ewing sarcoma is a malignancy of primitive cells, possibly of mesenchymal origin. It is probable that genetic perturbations other than *EWS-FLI1* cooperate with it to produce the tumor. Sequencing studies identified *STAG2* mutations in approximately 15% of cases in humans. In the present study, we hypothesize that loss of *Stag2* cooperates with *EWS-FLI1* in generating sarcomas derived from murine mesenchymal stem cells (MSCs).

**Methods:**

Mice bearing an inducible *EWS-FLI1* transgene were crossed to *p53*^*−/−*^ mice in pure C57/Bl6 background. MSCs were derived from the bone marrow of the mice. *EWS-FLI1* induction and *Stag2* knockdown were achieved in vitro by adenovirus-Cre and shRNA-bearing pGIPZ lentiviral infection, respectively. The cells were then treated with ionizing radiation to 10 Gy. Anchorage independent growth in vitro was assessed by soft agar assays. Cellular migration and invasion were evaluated by transwell assays. Cells were injected with Matrigel intramuscularly into C57/Bl6 mice to test for tumor formation.

**Results:**

Primary murine MSCs with the genotype *EWS-FLI1 p53*^*−/−*^ were resistant to transformation and did not form tumors in syngeneic mice without irradiation. *Stag2* inhibition increased the efficiency and speed of sarcoma formation significantly in irradiated *EWS-FLI1 p53*^*−/−*^ MSCs. The efficiency of tumor formation was 91% for cells in mice injected with *Stag2*-repressed cells and 22% for mice receiving cells without *Stag2* inhibition (*p* < .001). *Stag2* knockdown reduced survival of mice in Kaplan-Meier analysis (p < .001). It also increased MSC migration and invasion in vitro but did not affect proliferation rate or aneuploidy.

**Conclusion:**

Loss of *Stag2* has a synergistic effect with *EWS-FLI1* in the production of sarcomas from murine MSCs, but the mechanism may not relate to increased proliferation or chromosomal instability. Primary murine MSCs are resistant to transformation, and the combination of *p53* null mutation, *EWS-FLI1*, and *Stag2* inhibition does not confer immediate conversion of MSCs to sarcomas. Irradiation is necessary in this model, suggesting that perturbations of other genes beside *Stag2* and *p53* are likely to be essential in the development of *EWS-FLI1-*driven sarcomas from MSCs.

## Background

Ewing sarcoma is a malignancy of primitive cells that arises typically in young adolescents and adults [1, 2]. It is driven most frequently by the *EWS-FLI1* translocation, which fuses the *EWS* gene on chromosome 22 to the *FLI1* gene on chromosome 11 [3, 4]. The encoded oncoprotein recognizes specific transcriptional sequences via the DNA-binding domain of FLI1 and modulates target gene expression, but may be insufficient by itself to induce the disease. Other genetic mutations and the cellular context are likely to be important [5–9]. Recent studies have identified *STAG2* mutation as one of the most common associated anomalies in Ewing sarcoma, occurring in approximately 15% of tumor samples [10, 11]. However, the functional significance of this genetic perturbation remains to be elucidated.

The cohesin complex comprises 4 distinct proteins – SMC1, SMC3, RAD21, and either STAG1 or STAG2 [12–14]. It is required for proper sister chromatid segregation and therefore seems important for genomic stability [13, 15–18]. *STAG2* encodes the gene for stromal antigen 2 (SA2 or STAG2), which is more abundant than STAG1 in human cells [14]. Its mutational inactivation or loss of expression have been documented in a variety of solid and hematologic malignancies, including glioblastoma, lymphomas, colorectal, prostate, urothelial bladder cancers, and Ewing sarcoma [14, 17, 19–24].

In the present study, we sought to develop a system for investigating the potential role of cooperating genes that contribute to the development of Ewing sarcoma. Mesenchymal precursor cells have recently been used to model sarcomagenesis [25]. As the cell of origin of Ewing sarcomas may also be derived from a primitive mesenchymal cell, we felt a similar approach would be worth exploring. We previously developed a murine model in which *EWS-FLI1* expression could be conditionally activated through the expression of Cre recombinase [26]. In the current study, we isolate MSCs derived from these mice and re-inject them into syngeneic mice after genetic manipulations in cell culture. Using this new system, we present in vitro and in vivo data that support a synergistic effect between *Stag2* inhibition, *EWS-FLI1* expression, and *p53* mutation in the transformation of primary MSCs. The primary objective of the study is to determine whether *Stag2* down-regulation might cooperate with *EWS-FLI1* in the generation of sarcomas from MSCs.

## Methods

### Mice

**Fig. 1:**
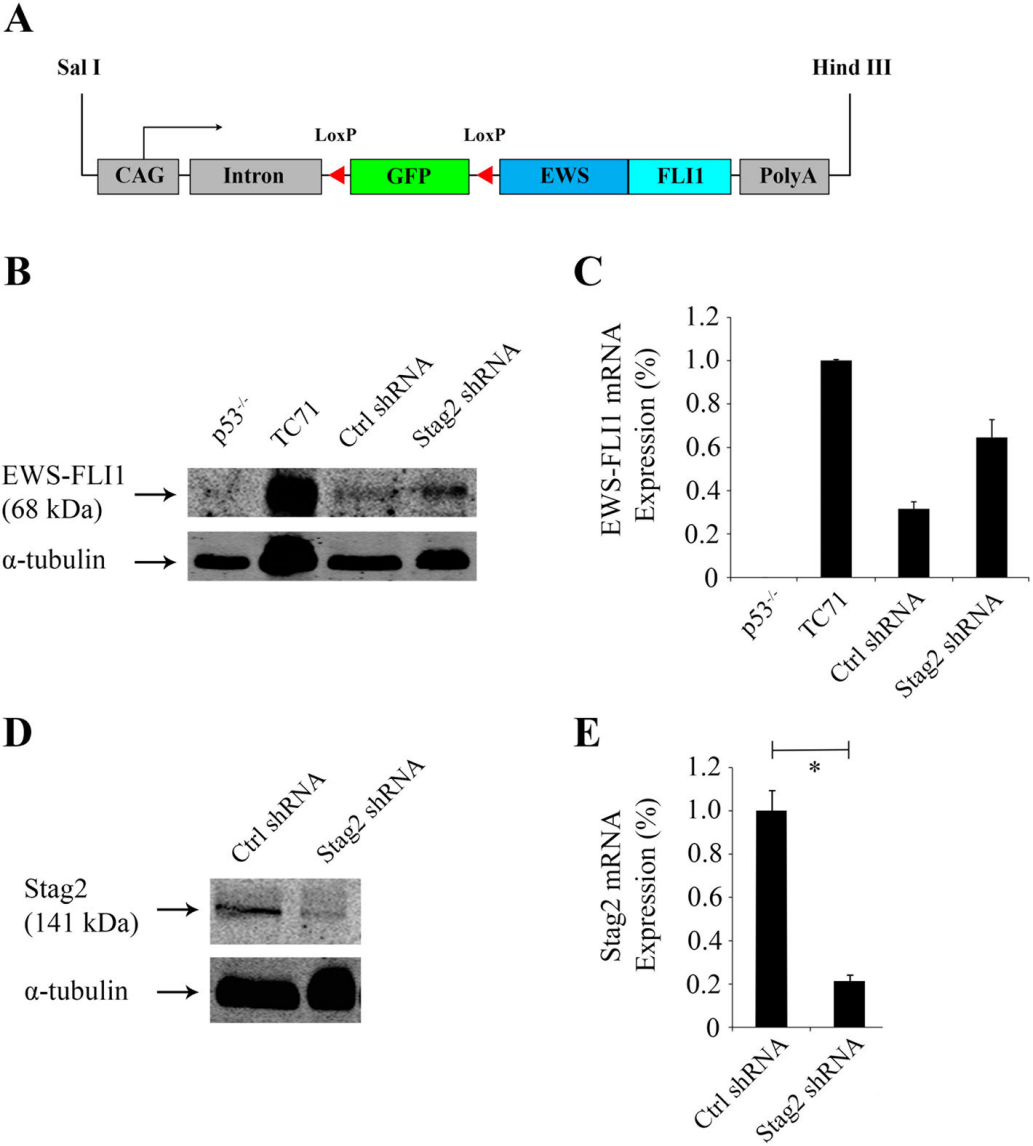
*EWS-FLI1* expression and knockdown of *Stag2* in MSCs. **a** Schematic diagram is shown for the *EWS-FLI1* transgene. Transcription (arrow) is driven by the CAG synthetic promoter, consisting of the chick β-actin core promoter with the cytomegalovirus immediate early enhancer and rabbit β-globin splice acceptor. LoxP sites flank the green fluorescent protein (GFP) gene. **b** Western blot with anti-FLI1 antibody shows *EWS-FLI1* expression in the Ewing sarcoma cell line TC71 carrying the Type 1 fusion (positive control) but not murine MSCs bearing the *p53* null mutation alone without *EWS-FLI1* (p53^−/−^, negative control). Positive expression of *EWS-FLI1* was observed in *EWS-FLI1 p53*^−/−^ MSCs after treatment with random control shRNA (“Ctrl shRNA” cells) and *EWS-FLI1 p53*^−/−^ MSCs after treatment with *Stag2* shRNA (“Stag2 shRNA” cells). Digital scanning of the Western blot showed that the level of protein expression of EWS-FLI1 (band intensity as a percentage of TC71) was 32% in Ctrl shRNA and 65% in Stag2 shRNA cells. **c** Quantitative RT-PCR, with *Rplp0* as the internal reference, confirms mRNA expression of *EWS-FLI1* in the same cells. **d** Stag2 shRNA cells show a decrease in expression of Stag2 compared to Ctrl shRNA cells on Western blot. **e** Quantitative RT-PCR, with *Rplp0* as the internal reference, showed that *Stag2* expression was reduced by 78% in Stag2 shRNA cells compared to Ctrl shRNA cells (*p* < .01)

All mice were maintained in C57/BL6J background. Transgenic mice with an inducible *EWS-FLI1* transgene [26] (Fig. 1) were backcrossed to pure C57/BL6J mice (The Jackson Laboratory, Bar Harbor, Maine, USA) at least 7 generations to obtain mice with > 99% C57/BL6J background. The *p53*^*−/−*^ mice were obtained in pure C57/BL6J background (The Jackson Laboratory, Bar Harbor, Maine, USA). All experiments were conducted in accordance with the National Institute of Health (NIH) Guide for the Care and Use of Laboratory Animals and were approved by the Institutional Animal Care and Use Committee at our institution (Project Identification Code: ACUF-00001165-RN00; approval date: November 19, 2014). Animals were housed in the institutional rodent colony facility in specific pathogen-free environment with sterilized cages, bedding, and food. Light/dark cycles, water, and temperature were regulated with automated control systems. Animals were checked on a regular basis by research and veterinary staff. Mice in distress were euthanized by CO_2_ ashpyxiation.

### Mesenchymal stem cell (MSC) isolation and culture

Tibial and femoral bones were flushed with phosphate buffered saline (PBS) to extract bone marrow that contained mesenchymal stem cells (MSCs). After washing the aspirate extensively in PBS, cells were cultured in Minimum Essential Medium alpha (MEMα) (Thermo Fisher Scientific, Waltham, MA, USA) supplemented with 15% fetal bovine serum (FBS, Gibco, New York, USA) and 1% penicillin-streptomycin (Sigma-Aldrich, Saint Louis, Missouri, USA). Cells were grown at 37 °C with 5% CO_2_. Non-adherent cells were discarded after 3 and 24 h. When confluent, the cultures were passaged by detaching with 0.25% trypsin-EDTA (Sigma-Aldrich, Saint Louis, Missouri, USA). Cells that continued to grow and propagate beyond 7 passages were used for subsequent studies.

### Induction of EWS-FLI1 expression

MSCs bearing the *EWS-FLI1* transgene were infected with adenovirus-Cre to remove the green fluorescent protein (GFP) stop cassette and induce expression of *EWS-FLI1*. Cells that did not lose GFP expression were removed by flow cytometry.

### Stag2 inhibition

*Stag2* short hairpin RNA (shRNA) sequences were generated by cloning into the lentiviral pGIPZ vector (Open Biosystems/GE Dharmacon, Lafayette, Colorado, USA). After testing several shRNAs, the clone bearing the target sequence 5’AGAACTTCTTCACTACTCT3’ was chosen for subsequent experiments. A scrambled nonsense control (Ctrl) shRNA with the target sequence 5’CTTACTCTCGCCCAAGCGAGAT3’ was cloned into the same lentiviral shuttle and used as a negative control. The constructs were transfected into human embryonic kidney (HEK 293) cells and the media was collected to obtain lentivirus. For infection of MSCs, the cells were seeded into 10 cm plates at a density of 2 × 10^6^ cells/plate, and incubated with media containing lentivirus. Antibiotic-resistant cells were selected by puromycin (Invitrogen, Carlsbad, California, USA) and green fluorescence of the cells was checked by microscopy and flow cytometry.

### Western blot

Total protein from cells was extracted in radioimmunoprecipitation assay (RIPA) lysis buffer and quantified using the bicinchoninic acid (BCA, Thermo-Fisher) test. Twenty to 30 μg of protein from each sample were separated by 10% sodium dodecyl sulfate (SDS) polyacrylamide gel electrophoresis and electroblotted onto polyvinylidene difluoride (PVDF) membranes (Millipore, Bedford, Massachusetts, USA). The membranes were blocked with 5% non-fat milk for 1 h, washed 3 times with Tris-buffered saline containing 1% Tween 20 (TBST) at room temperature and then incubated overnight at 4 °C with the antibody. These included the polyclonal rabbit antibodies against *FLI1* (1:250 dilution, Santa Cruz Biotechnology, Dallas, Texas, USA) and the monoclonal mouse antibody against *Stag2* (1:500 dilution, Santa Cruz Biotechnology, Dallas, Texas, USA). After washing with TBST, membranes were incubated with secondary antibodies at room temperature for 1 h (goat anti-rabbit and goat anti-mouse IgG, 1:10,000 dilution, Jackson ImmunoResearch Laboratories). After washing with TBST, immunoreactivity was visualized by enhanced chemiluminescence reagents (Millipore, Bedford, Massachusetts, USA). MSCs from normal mice were used as negative controls, while the human tumor cell line TC71 was used as positive controls for *EWS-FLI*.

### RNA extraction and RT-PCR

RNA expression levels of *EWS-FLI1*, *Stag2*, *Smc1a*, *Smc1b*, *Smc3*, and *Rad21* were assayed by quantitative RT-PCR. Total RNA was extracted by the RNA Extraction Kit (QIAgen, Hilden, Germany). Quantity and quality of RNA were confirmed by a NanoDrop 2000 Spectrophotometer (Thermo Fisher Scientific, Wilmington, Detroit, USA). For mRNA expression, cDNA was obtained by using the iScript Reverse Transcription Supermix for RT-PCR (Bio-Rad Laboratories Inc., Hercules, California, USA) with oligodT_15_ primers. Mouse *Rplp0* mRNA was used as a reference gene to normalize for mRNA expression. RT-PCR was performed using SYBR® Green PCR Master Mix and the ABI 7500 Fast instrument (Thermo Fisher Scientific, Wilmington, DE USA). Data were calculated relative to *Rplp0*, based on calculations of 2^−△Ct^ where −△Ct = Ct (Target) – Ct (Reference). Fold change was presented by the 2^−△△Ct^ method. Sequences of primers for RT-PCR are listed in Table 1.

**Table 1:** Primer sequences used for *EWS-FLI1* and *Stag2* detection by RT-PCR

*EWS-FLI1*	Forward	5′-GCTCCAAGTCAATATAGCCAACAG-3′
	Reverse	5′-AAGCTCCTCTTCTGACTGAGTCATAA-3′
S*tag2*	Forward	5′-CAGCTGAATGTCATCCTCCC-3’
	Reverse	5′-GCAAACAGCTCAGTGATTCTTG-3’
S*mc1a*	Forward	5′-GTTTACCGCCATCATTGGACC-3’
	Reverse	5′-GGTCCCGCAGGGTCTTTAC-3’
S*mc1b*	Forward	5′-CGCAAGAAGGTTTTGGCTGTT-3’
	Reverse	5′-CCAATGACCCCAGCACAAGA-3’
S*mc3*	Forward	5′-GATGAAGGAGAAGGGAGTGGT-3’
	Reverse	5′-ATCAGAGCAAGGGCTACCAAG-3’
R*ad21*	Forward	5′-CTACTGAAGCTCTTTACACGCTGTC-3’
	Reverse	5′-ATGCTGCTGTTGCTGGTCCTC-3’

### Cellular growth in vitro

The proliferation rate of cells grown in monolayer culture was measured in the following manner. After plating 5 × 10^4^ cells into dishes, cells were detached at regular intervals with trypsin-EDTA, diluted with 5 mL of MEMα, and counted in triplicate with a hemocytometer.

Anchorage-independent cellular growth was assessed by soft agar colony formation. Approximately 5 × 10^3^ cells were plated in 1 ml of 0.3% (weight/volume) noble agar (Sigma-Aldrich, Saint Louis, Missouri, USA) in culture medium on a solidified basal layer agar (1.5 ml of 0.5% agar in medium) per 35 mm culture plates. After 30 min at room temperature, the top agar solidified, and the plates were returned to the incubator at 37 °C. We refreshed media by adding 150 μL of MEMα to the plates weekly. After 21 days, 200 μL of 3-[4,5-dimethylthiazole-2-yl]-2,5-diphenyltetrazolium bromide (MTT) were added to stain the cells. The MDAMB231 breast cancer cell line was used as a positive control. The NIH ImageJ software was used to quantify colony formation in soft-agar. Statistical calculations were based upon the mean number of colonies per plate and the mean size of the colonies. All samples were tested in triplicate.

### Transwell cell migration and invasion assays

Cell migration was assessed by 8 μm-pore transwell polycarbonate membranes (Corning Inc., New York, USA). In the upper chamber, 1.5 × 10^4^ cells (per sample) were seeded with 200 μL of serum-free MEMα. In the lower chamber, 500 μL of MEMα containing 15% FBS was added for chemotaxis. After incubating for 24 h at 37 °C, the migratory cells were fixed with 2% methanol for 5 min and stained with crystal violet. Photographs of four randomly selected fields were then taken and cell numbers counted under a microscope at 200x magnification. Each test was performed in triplicate. The cellular invasion assay was performed in similar fashion with the modification that membranes were coated with 100 μL of 1 mg/mL Matrigel (Corning Inc., New York, USA) diluted in PBS. Cell counting was performed as described above.

### Flow cytometry

To analyze DNA content and determine the proportion of cells in the phases of the cell cycle, growing cells were trypsinized and collected by centrifugation. Cell pellets were gently suspended in 5 mL of 95% ethanol for 30 min at room temperature for fixation. Cells were centrifuged and resuspended in 1 mL PBS mixed with 50 μg/ml propidium iodide (Invitrogen, Carlsbad, California, USA). RNA was removed by incubation for 30 min with 80 μL of 1 mg/mL RNAse A (Roche Diagnostics, Indianapolis, USA). After filtration through a 50 μm membrane, cells were analyzed by flow cytometry in a Gallios 561 instrument (Beckman Coulter Inc., Indianapolis, USA). To induce apoptosis prior to flow cytometry, cells were seeded at a density of 1 × 10^6^ per cell plate and placed in serum-starvation medium containing 0.01% FBS. Analyses were performed at 24 and 48 h after induction of apoptosis.

### Tumor formation in mice

Cultured cells which were 80–90% confluent were detached by trypsin-EDTA, counted in a hemocytometer, and collected by centrifugation. After washing, resuspending in PBS, and chilling on ice, liquid cold Matrigel was added (1:2 volume ratio to PBS) to achieve a final cell concentration of 1-2 × 10^7^ cells/mL. The cell suspension was kept on ice to avoid the premature solidification of the Matrigel. Intramuscular injection into the quadriceps muscle of healthy, normal adult C57/Bl6 mice at 3–6 months of age (baseline weight 20–30 g) was performed with an insulin syringe to deliver 1 × 10^6^ cells per site. All animals tolerated the injection well, and there were no adverse events in the form of deaths or infections from the injections. The primary experimental outcome was the formation of tumor at the injected site, and the secondary experimental outcome was the latency in time to tumor formation. Animals were randomly allocated to study groups. The sample sizes were estimated by power analyses to achieve an 80% probability of detecting a 50% difference in the proportion of mice forming tumors. Mice were monitored daily for tumor formation. Mice were sacrificed by CO2 asphyxiation before they showed signs of distress and before tumors exceeded 1.0 cm in size.

### Statistical tests

Quantitative analyses were performed with SPSS® version 24 (IBM Corp., Armonk, New York, USA). The student’s T-test was used to compare the mean numbers and sizes of colonies between the different cell types. We employed the chi square test to detect a difference in tumor formation between the injected mice. Growth curves were compared using the mixed design analysis of variance model (split-plot ANOVA). Kaplan-Meier survival analysis with the log-rank test was used to evaluate survival related to tumor development in mice. A *p*-value less than 0.05 was accepted as statistically significant.

## Results

### Inhibition of Stag2 in MSCs bearing the genotype p53^−/−^ EWS-FLI1

Starting with an *EWS-FLI1* transgenic mouse bearing a construct that allows for conditional expression [26], we crossed to *p53*^*−/−*^ null mice to obtain *EWS-FLI1 p53*^−/−^ mice in a pure C57/Bl6 background. From these mice, we isolated and cultured MSCs from the bone marrow of the femur and tibia. Adenovirus-Cre infection of the cells activated expression of the *EWS-FLI1* gene by removal of the floxed GFP-containing reporter cassette (Fig. 1a). Cells subsequently underwent fluorescence-activated cell sorting (FACS) to isolate the GFP negative, *EWS-FLI1 p53*^*−/−*^ cell population. Western blot and RT-PCR confirmed protein and mRNA expression of *EWS-FLI1*, respectively (Fig. 1a, b). In an in vivo experiment, injection of the *EWS-FLI1 p53*^−/−^ MSCs into 12 syngeneic pure C57/Bl6 mice produced no tumors.

Since the combination of *p53*-null mutation and *EWS-FLI1* was insufficient to transform the cells, we introduced *Stag2* knockdown as an additional genetic event in the cells. Using a retroviral GIPZ construct, we expressed *Stag2* shRNA or random control (Ctrl) shRNA in the *EWS-FLI1 p53*^−/−^ MSCs (henceforth designated “Stag2 shRNA” and “Ctrl shRNA” cells, respectively). Western blot confirmed a reduction in Stag2 expression in the Stag2 shRNA cells (Fig. 1c). RT-PCR showed that *Stag2* mRNA expression was decreased by 78% in Stag2 shRNA compared to Ctrl shRNA cells (Fig. 1d).

### Stag2 inhibition does not increase chromosomal aberrations

**Fig. 2:**
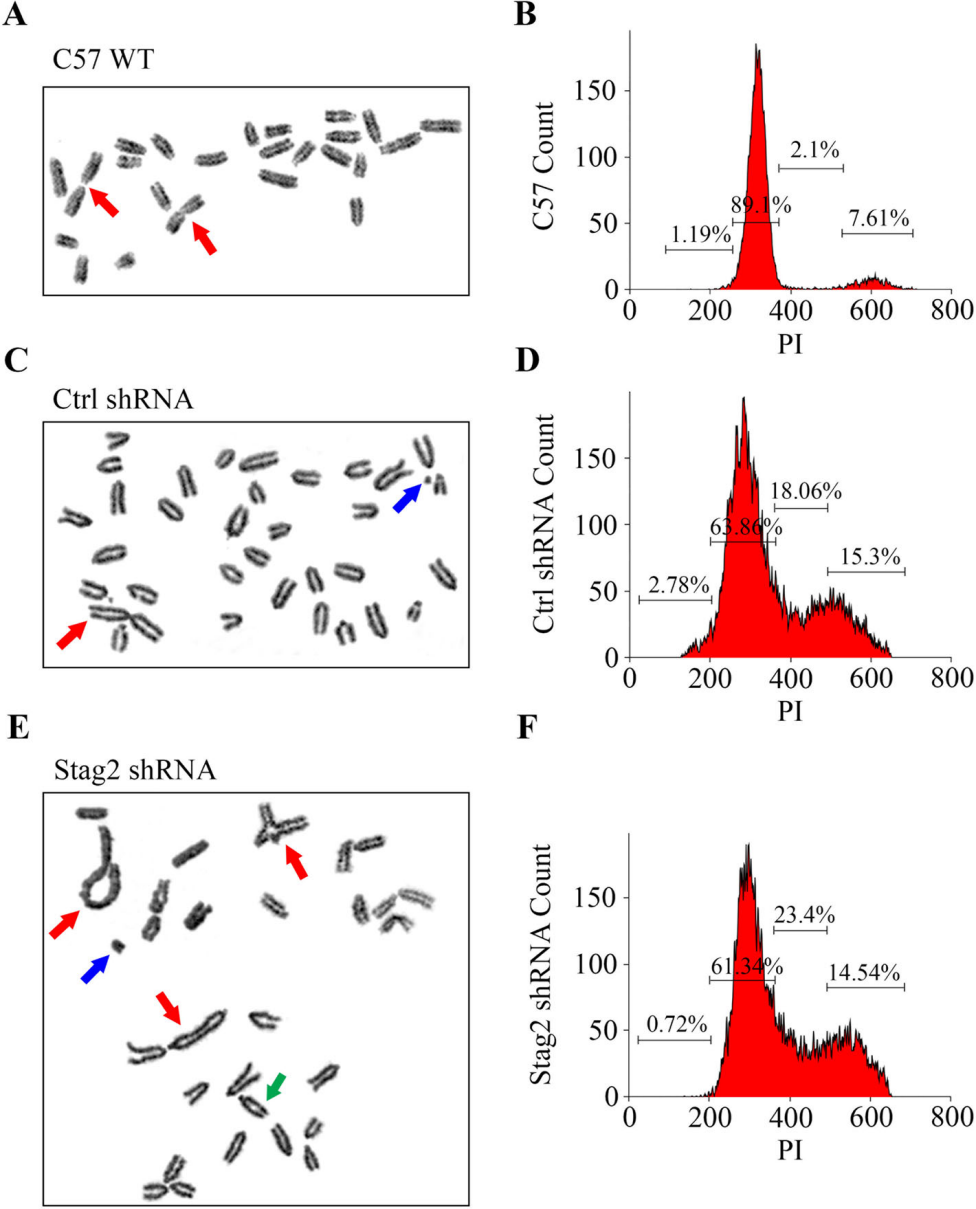
Chromosomal abnormalities. Metaphase chromosomal spreads were prepared from MSCs with the following genotypes **a** pure wild-type C57/Bl6 (C57 WT) cells; **c** EWS-FLI1 p53−/− cells expressing random control shRNA (Ctrl shRNA cells); and **e** EWS-FLI1 p53−/− cells expressing Stag2 shRNA (Stag2 shRNA cells). Examination of 125 metaphase spreads showed more abnormal metaphases for Ctrl shRNA and Stag2 shRNA cells compared to C57 WT cells. Ctrl shRNA and Stag2 shRNA cells exhibited frequent non-reciprocal translocations (red arrows), chromosomal fragments (blue arrows) and chromosomal breaks (green arrows). However, there was no significant difference between Ctrl shRNA and Stag2 shRNA cells in terms of percentage of aberrant metaphases (34% vs. 34%, respectively), chromosomal breaks (18% vs. 16%, respectively), and chromosomal fusions/translocations (24% vs. 24%). **b** The cell cycle distribution of C57/Bl6 WT cells stained with propidium iodide (PI) showed 89.1% of cells in G0-G1, 2.1% in S, and 7.6% in G2-M phases. Cell cycle distribution of Ctrl shRNA cells **d** and Stag2 shRNA cells **f** showed a higher fraction of non-G0-G1 cells compared to the control C57 WT cells. The cell cycle distribution of Ctrl shRNA cells was not statistically different compared to Stag2 shRNA cells

Using metaphase chromosomal spreads to examine the effect of *Stag2* inhibition, we noted greater aberrations in Ctrl shRNA and Stag2 shRNA cells (both of which express *EWS-FLI1* and carry a *p53*^−/−^ null mutation) when compared with MSCs derived from normal wild-type C57/Bl6 mice. The percentage of abnormal metaphase spreads, chromosomal breaks, fusions, and cells with abnormal ploidy was not statistically different between Ctrl shRNA and Stag2 shRNA cells (Fig. 2).

The distribution of cells in the phases of the cell cycle was not different between Ctrl shRNA and Stag2 shRNA cells (Fig. 2; Table 2). Both had a greater proportion of non-G0-G1 cells compared to C57/Bl6 cells. Flow cytometry was also used to gain a quantitative measure of the DNA content of cells and the fraction of euploid cells. Cell cycle analysis post-apoptosis induction did not reveal a significant difference in the distribution of the phases between Stag2 shRNA cells and Ctrl shRNA cells, at both 24 and 48 h after serum starvation (*p* > 0.05).

**Table 2:** Cell cycle analysis distribution between Ctrl shRNA and Stag2 shRNA cells

	Ctrl shRNA		Stag2 shRNA		Association
	Mean	95% CI	Mean	95% CI	p-value
Sub-G0	1.79	0.38–3.2	1.73	0–4.33	0.97
G0-G1	62.24	56.93–67.54	56.17	48.8–63.55	0.25
S	21.46	13.56–29.36	22.3	20.67–23.93	0.8
G2	14.66	13.45–15.87	19.83	14.22–25.44	0.14

### Loss of Stag2 cooperates with EWS-FLI1 and p53 mutation to generate sarcomas after irradiation

Anchorage-independent growth, as determined by colony formation in soft agar, did not occur in *EWS-FLI1 p53*^*−/−*^ MSCs, even after *Stag2* knockdown. Furthermore, the same cells did not form tumors in vivo after injection into syngeneic C57/Bl6 normal mice. Of the 16 mice injected with Stag2 shRNA cells (*EWS-FLI1 p53*^−/−^ MSCs expressing *Stag2* shRNA), none exhibited any tumor formation at 12 months follow-up. The same results were obtained with Ctrl shRNA cells (*EWS-FLI1 p53*^−/−^ MSCs expressing control shRNA).

**Fig. 3:**
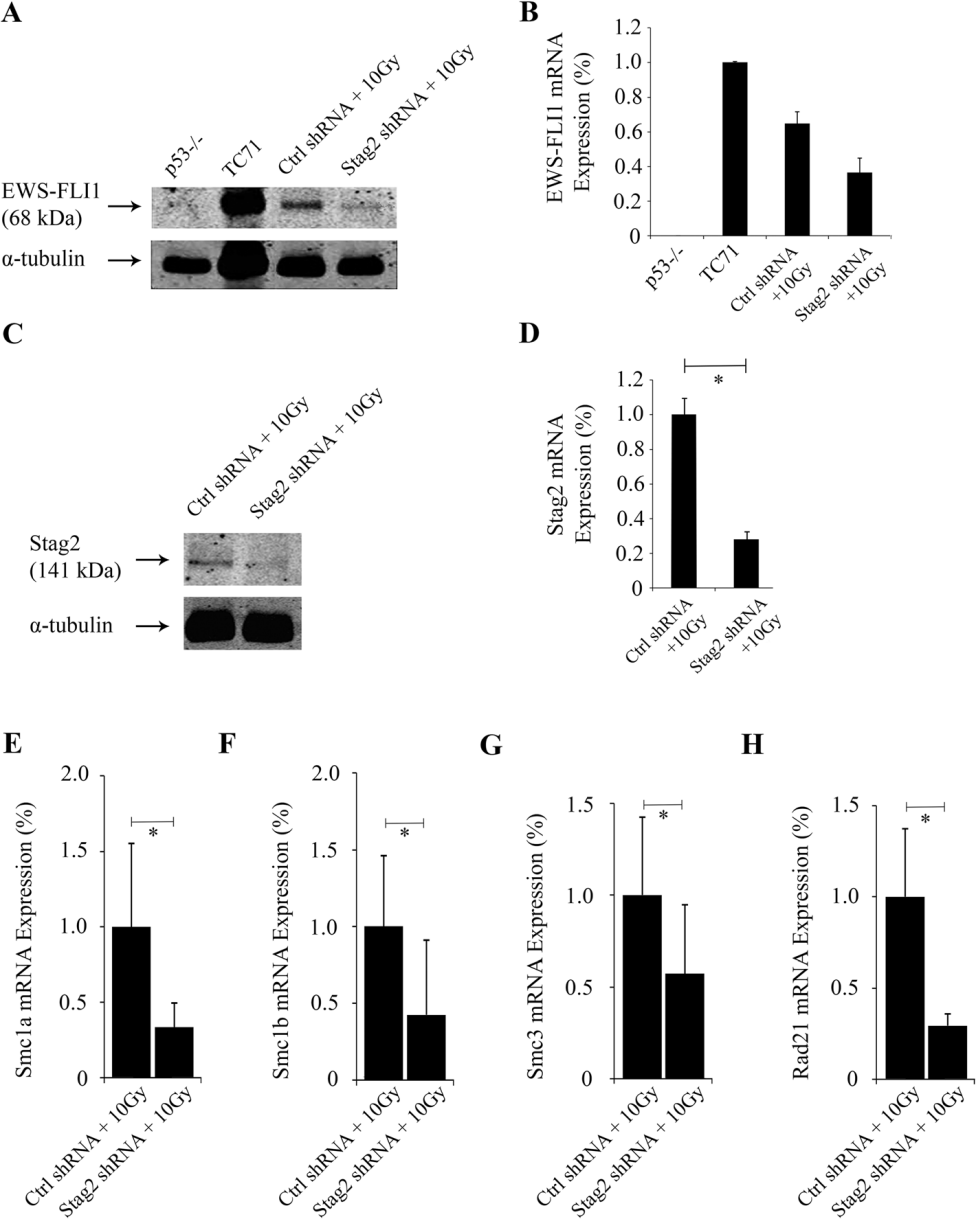
Verification of *EWS-FLI1* expression and *Stag2* knockdown after irradiation of MSCs*.* **a** Western blot with anti-FLI1 antibody shows EWS-FLI1 expression in the Ewing sarcoma cell line TC71 (positive control) but not *p53*^*−/−*^ cells without EWS-FLI1 (negative control). Both Ctrl shRNA+10Gy and Stag2 shRNA+10Gy irradiated cells showed positive EWS-FLI1 expression. Digital scanning of the Western blot showed that the level of protein expression of EWS-FLI1 (band intensity as a percentage of TC71) was 64.9% in Ctrl shRNA+10Gy and 36.5% in Stag2 shRNA+10Gy cells. **b** Quantitative RT-PCR, with *Rplp0* as the internal reference, confirms mRNA expression of *EWS-FLI1* in the same cells. **c** Western blot for *Stag2* shows diminished expression in Stag2 shRNA+10Gy compared to Ctrl shRNA+10Gy cells. **d** Quantitative RT-PCR, with *Rplp0* as the internal reference, showed that *Stag2* expression was reduced by 63% in Stag2 shRNA+10Gy compared to Ctrl shRNA+10Gy cells (p < .01). **e–h** For the genes of the cohesin complex that are coordinately expressed with *Stag2*, the expression levels of *Smc1a* **e**, *Smc1b* **f**, *Smc3* **g**, and *Rad21* **h** were reduced by 66, 57, 43, and 71%, respectively, in Stag2 shRNA+10Gy cells compared to Ctrl shRNA+10Gy cells (p < .01). Values were normalized to *Rplp0* expression, and the level of gene expression in Ctrl shRNA+10Gy cells was set as the reference baseline

The findings underscored a certain resistance of primary murine MSCs towards neoplastic transformation. The simultaneous presence of the three induced genetic changes (*p53* null mutation, *EWS-FLI1* expression, and *Stag2* inhibition) was insufficient to produce immediate, full neoplastic transformation. We therefore treated MSCs with 10 Gy of ionizing radiation to induce further genetic perturbations. Radiation-treated Stag2 shRNA cells were designated “Stag2 shRNA+10Gy”, whereas radiation-treated Ctrl shRNA cells were designated “Ctrl shRNA+10Gy”. Western blot and RT-PCR confirmed the continued expression of *EWS-FLI1* in the irradiated cells as well as decreased expression of Stag2 after knockdown (Fig. 3a–d). Furthermore, the mRNA expression of the cohesin complex genes *Smc1a*, *Smc1b, Smc3,* and *Rad21*, which are coordinately expressed with *Stag2* [27, 28], was diminished in Stag2 shRNA+10Gy cells compared to Ctrl shRNA+10Gy cells (Fig. 3e–h). Together, the results indicated that the Stag2 shRNA+10Gy cells had both the intended genotype as well as expression pattern with respect to the *EWS-FLI1* and *Stag2* genes.

**Fig. 4:**
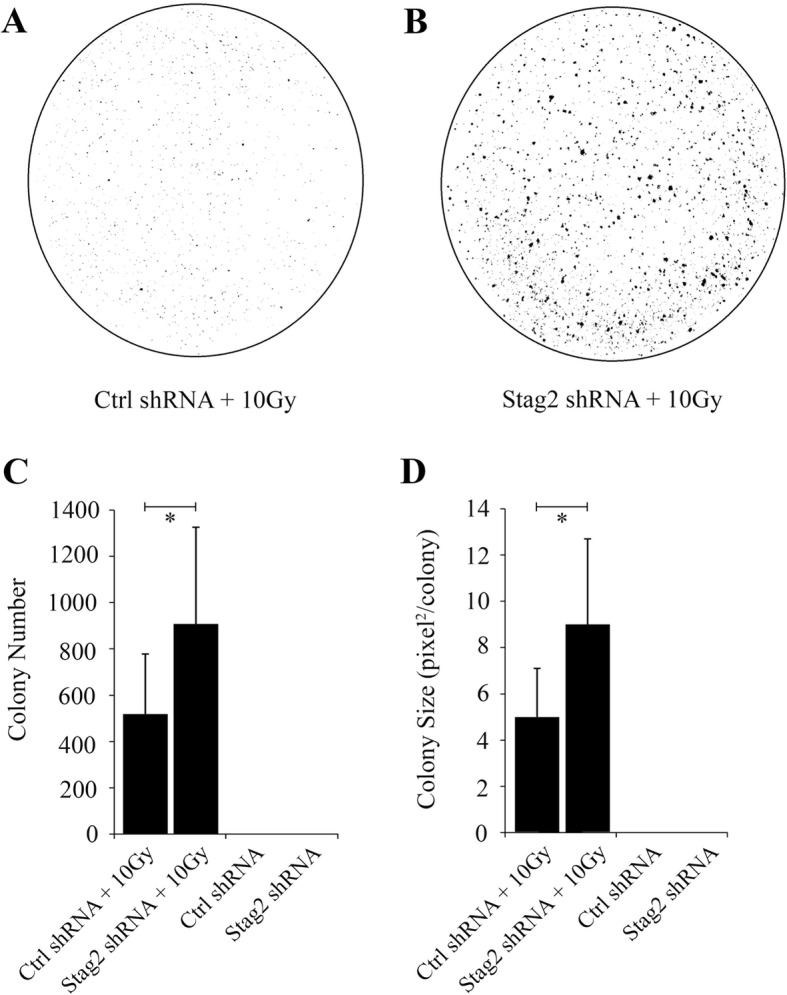
Anchorage-independent growth in soft agar after Stag2 knockdown. Representative plates are shown for **a** Ctrl shRNA+10Gy cells and **b** Stag2 shRNA+10Gy cells. **c** The mean number of colonies per plate was 908 (95% CI 744–1072) for Stag2 shRNA+10Gy cells and 520 (95% CI 422–618) for Ctrl shRNA+10Gy cells (*p* < .001). **d** Digital image analysis to determine colony size by pixels showed a mean size of 4.6 pixels/colony (95% CI 3.9–5.4) for Ctrl shRNA+10Gy cells compared to 8.8 pixels/colony (95% CI 7.4–10.3) for shRNA+10Gy cells (p < .001). Assays were done in triplicate

In soft agar anchorage-independent growth assays, there were significantly more colonies in the Stag2 shRNA+10Gy cultures compared to Ctrl shRNA+10Gy cultures (Fig. 4a–c). Mean colony size was also significantly greater for Stag2 shRNA+10Gy cells (Fig. 4d).

**Fig. 5:**
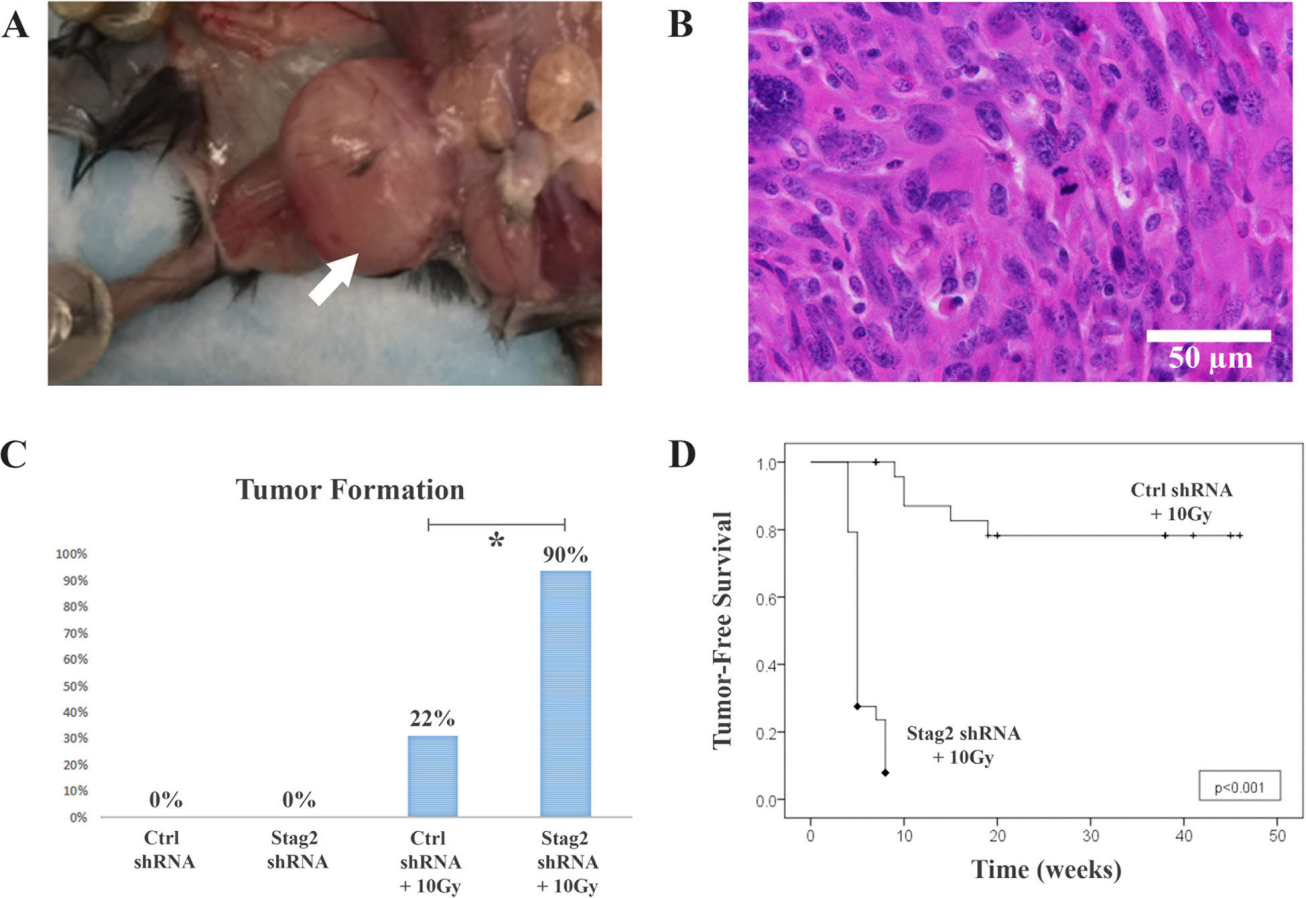
Formation of sarcomas after injection of mice with MSCs in Matrigel carrier. **a** Tumor formation (arrow) in the quadriceps muscle is shown after injection of 1 × 10^6^ Stag2 shRNA+10Gy cells (irradiated MSCs with *Stag2* knockdown, *EWS-FLI1* expression, and *p53*^−/−^ null mutation). **b** Histopathology shows a pleomorphic spindle cell sarcoma with frequent mitotic figures. **c** The rate of tumor formation is significantly higher for Stag2 shRNA+10Gy compared to Ctrl shRNA+10Gy cells (*p* < .001). **d** Kaplan-Meier survival is significantly shorter for mice injected with Stag2 shRNA+10Gy compared to Ctrl shRNA+10Gy cells (*p* < .001)

After intra-muscular injection of cells, 5 of 23 mice (22%) with Ctrl shRNA+10Gy cells developed tumors, whereas 19 of 21 mice (91%) injected with Stag2 shRNA+10Gy cells developed tumors (*p* < .001, Fig. 5). Mean time for tumor development was 1.2 months (range 0.8–1.8 months) for Stag2 shRNA+10Gy cells with *Stag2* knockdown and 3.1 months (range 1.6–5.5 months) for Ctrl shRNA+10Gy cells without *Stag2* knockdown (p < .001). All tumors were undifferentiated pleomorphic sarcomas (Fig. 5). In a parallel control experiment, *p53*^*−/−*^ null MSCs without *EWS-FLI1* were treated with *Stag2* knockdown and 10 Gy radiation. Of the 14 mice injected with the cells, none developed tumors. Taken together, the results indicate that synergy exists between *Stag2* inhibition, *p53* mutation, and *EWS-FLI1* expression in the process of sarcomagenesis.

### Stag2 inhibition increases invasion and migration but not proliferation

**Fig. 6:**
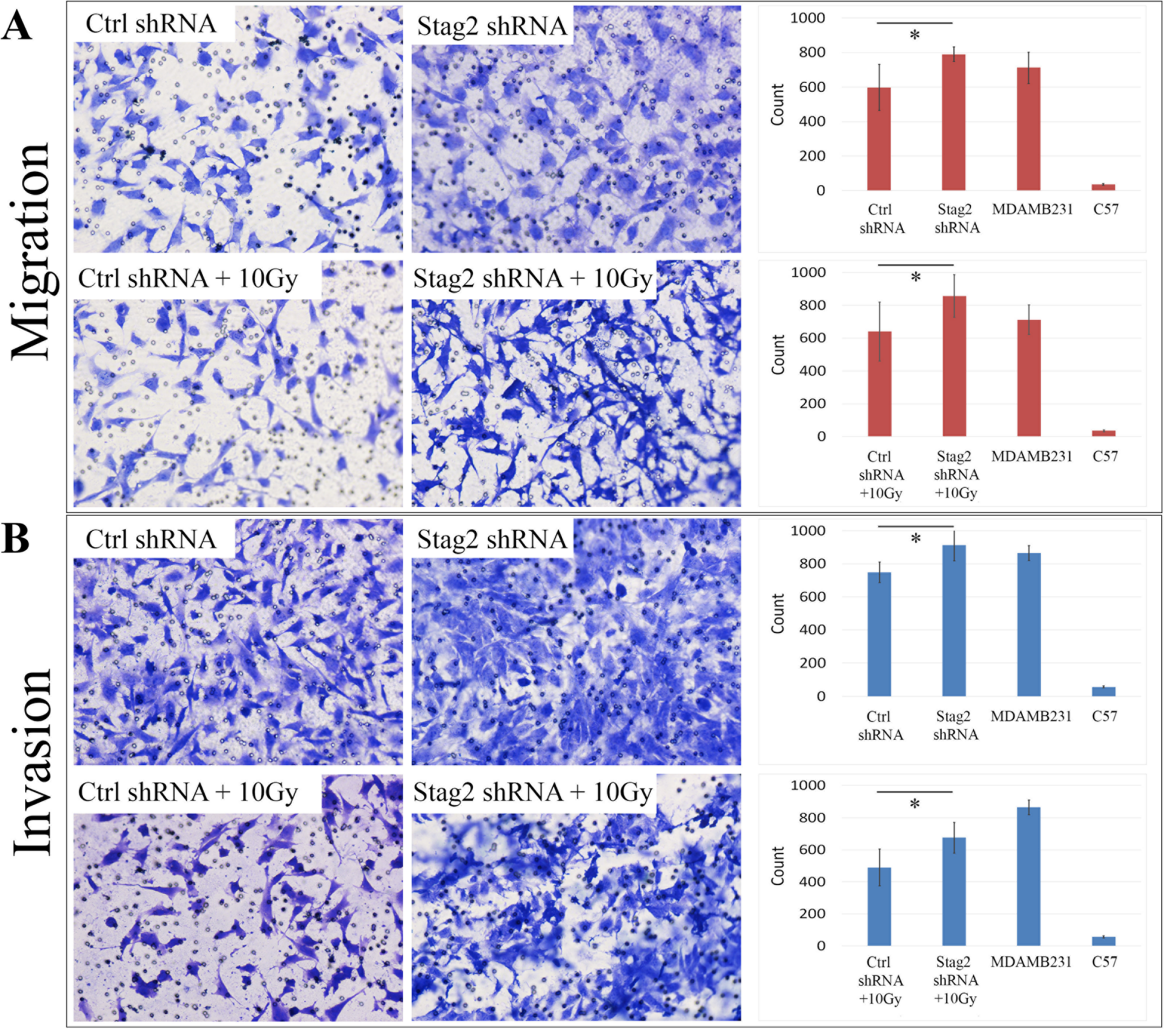
Migration and invasion assays. Transwell migration **a** and invasion **b** assays are depicted. A graph with a quantitative analysis is shown for each pair of cells. Normal C57/Bl6 MSCs were used as negative controls, whereas the breast cancer cell line MDAMB231 was the positive control. All assays were done in triplicate. Statistical significance is marked with an asterisk “*”. **a** For non-irradiated cells in the migration assay, we found that the mean number of migratory cells per plate was 597 (95% CI 497–696) for Ctrl shRNA cells compared to 789 (95% CI 759–818) for Stag2 shRNA cells (*p* = .004). For radiated cells, mean number of migratory cells per plate was 640 (95% CI 538–742) for Ctrl shRNA+10Gy migratory cells per plate compared to 857 (95% CI 785–929) for Stag2 shRNA+10Gy cells (*p* = .002). **b** For non-irradiated cells in the invasion assay, the mean number of invasive cells per plate was 749 (95% CI 704–794) for Ctrl shRNA compared to 914 (95% CI 831–996) for Stag2 shRNA cells (*p* = .006). For the radiated cells, the mean number of invasive cells per plate was 542 (95% CI 4907–594) for Ctrl shRNA+10Gy compared to 676 (95% CI 601–751) for Stag2 shRNA+10Gy cells (*p* = .008)

To determine whether loss of *Stag2* might enable MSCs to acquire certain properties of transformed cells, we performed Transwell migration and invasion assays. Migration increased in Stag2 shRNA and Stag2 shRNA+10Gy cells compared to Ctrl shRNA and Ctrl shRNA+10Gy cells, respectively (Fig. 6a). Similarly, inhibition of *Stag2* increased the invasive properties of MSCs compared to cells receiving control shRNA (Fig. 6b).

**Fig. 7:**
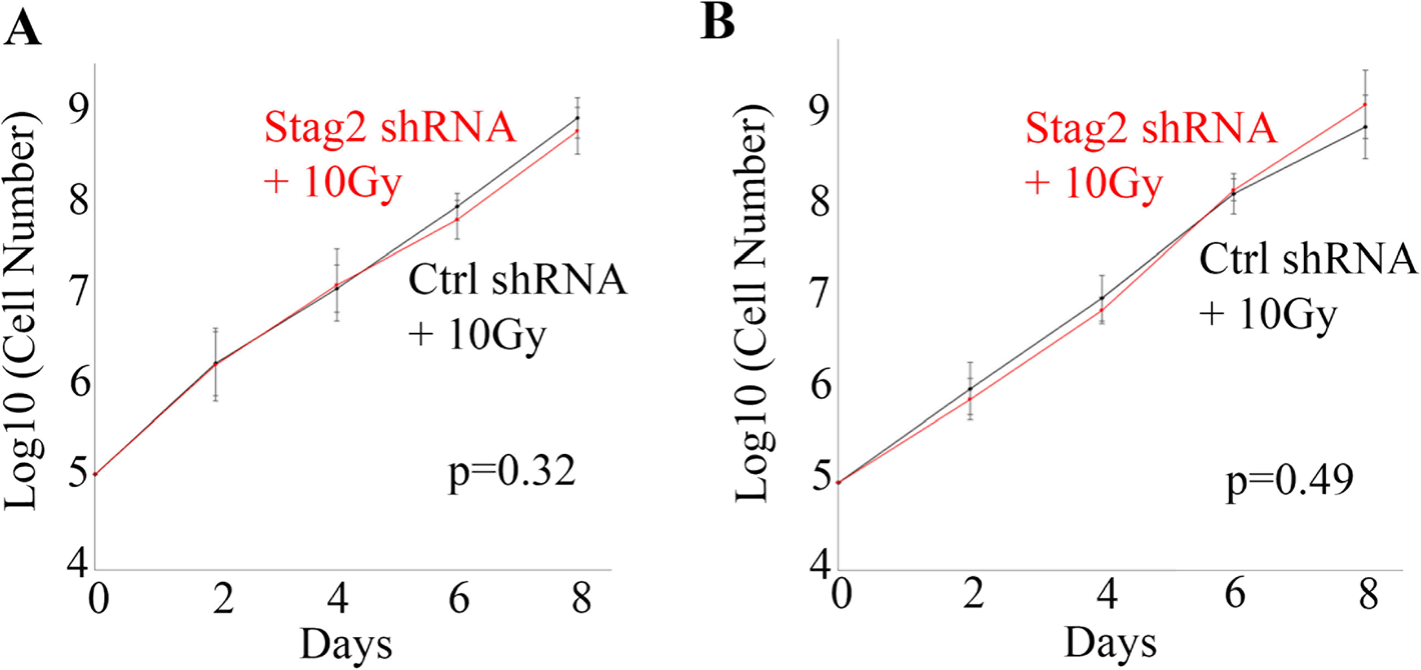
Proliferation rate in cell culture. Comparison of anchorage-dependent growth on plastic plates did not show a significant difference in growth rate between **a** Ctrl shRNA and Stag2 shRNA cells; and **b** Ctrl shRNA+10Gy and Stag2 shRNA+10Gy cells

*Stag2* knockdown did not have an appreciable effect on the proliferative rate of cells. In two-dimensional monolayer cultures, the growth curve of Ctrl shRNA cells was not different from Stag2 shRNA cells (Fig. 7a). Similarly, the growth curve of Ctrl shRNA+10Gy cells was not different from Stag2 shRNA+10Gy cells (Fig. 7b).

## Discussion

Ewing sarcoma is driven by an *EWS-ETS* translocation, which fuses the *EWS* gene on chromosome 22 to one of the *ETS* family member of genes, most commonly *FLI1* on chromosome 11 [29–33]. The fusion gene seems necessary for this type of tumor to develop, but it is not clear yet whether perturbations of other genes may also be important and how these genetic changes work together to produce a neoplastic cell. In our previous murine model, we found that conditional expression of *EWS-FLI1* alone in the limb bud did not produce sarcomas in mice, but loss of *p53* together with induction of *EWS-FLI1* accelerated sarcoma formation [26]. A number of genetic mutations, including *p53*, have been identified in Ewing sarcoma from sequencing studies, but none of these has been shown to be consistently present in a majority of patients [34–39]. Nevertheless, it is intriguing that approximately 15% of Ewing sarcoma samples exhibited mutations in the *STAG2* gene, making it one of the most commonly mutated genes in the disease [10, 11]. In this study, we explored the question of whether loss of *Stag2* might also cooperate with *EWS-FLI1* and *p53* loss in sarcomagenesis.

We used MSCs from genetically modified mice bearing the silent *EWS-FLI1* gene as the starting material for this study. While the true cell of origin of Ewing sarcoma is still a matter of debate, there is some evidence that it may be derived from primitive mesenchymal cells that have multi-potential capacity for differentiation [40–45]. Several studies have generated sarcomas in mice by expressing *EWS-FLI1* in MSCs [46–48]. One experimental advantage of using MSCs is that they are relatively easy to grow and manipulate genetically in vitro.

We found that primary MSCs from C57/Bl6 mice did not easily transform into sarcomas. In fact, the combination of *p53* null mutation, *EWS-FLI1* expression, and *Stag2* inhibition did not immediately confer tumor-forming capability to the cells. This observation indicated that additional genetic mutations or alterations of cellular state were necessary to achieve transformation. Indeed, upon the administration of 10 Gy radiation, the cells became tumorigenic, forming tumors quickly in nearly all mice. In these experiments, we observed that without *Stag2* loss, the combination of *p53* null mutation and *EWS-FLI1* expression with 10 Gy radiation also produced tumors, albeit with reduced efficiency and greater latency. Thus, we concluded that *Stag2* knockdown had a synergistic effect with *EWS-FLI1* in the production of sarcomas.

Other researchers have reported that *EWS-FLI1* alone is capable of transforming murine MSCs [46]. While this is fairly compelling data that *EWS-FLI1* can act as a driver mutation for sarcoma formation, it is important to recognize that a long time occurred between induction of the gene and tumor formation, during which additional mutations or changes in gene expression could occur. These additional events are of particular interest to us in our current line of investigation. In the previous paper, the efficiency of tumor formation increased with passage of cells in culture in vitro and passage of tumors in mice, consistent with the notion that additional genetic or epigenetic events accrued over time [46]. Furthermore, it may be relevant to point out that there are notable differences in the experimental systems. Our cells expressed an *EWS-FLI1* transgene, which had been stably integrated into the genome, whereas the previous investigators used a retroviral transfection technique to express *EWS-FLI1*. Additionally, their cells were injected into the renal capsule of severe combined immunodeficient mice whereas our cells were injected intramuscularly into syngeneic immunocompetent mice. These experimental differences may help explain why we observed a certain resistance of murine MSCs towards transformation.

The main finding that we would stress in the current study is that both *p53* mutation and *Stag2* loss could accelerate tumorigenesis with *EWS-FLI1*. This finding is compatible with prior studies showing the ability of *EWS-FLI1* to transform murine MSCs [46] and work showing that multiple mutations are necessary to transform MSCs [49].

Researchers have tried to decipher the mechanism by which loss of *STAG2* promotes tumorigenesis. As part of the cohesin complex that regulates chromatid segregation, STAG2 was initially believed to prevent aneuploidy [27]. However, recent studies did not find increased aneuploidy with *STAG2* loss [10, 11, 24, 28, 50]. In our study, we did not see an obvious increase in chromosomal instability or aneuploidy with *Stag2* depletion, but a subtle effect might have been overshadowed by the dominating effect of a *p53* null mutation. Theoretically, *EWS-FLI1* might also contribute to chromosomal rearrangements, but sequencing data in human tumors indicate that mutations are relatively uncommon in Ewing sarcoma, so that most of the changes would be attributed to *p53* null mutation [10, 11, 51, 52].

It is possible that *Stag2* loss affects DNA repair in different ways other than regulation of chromatid segregation [53]. Stag2 is essential for replication fork progression [54]. Stag2 binds to single strand DNA and double strand breaks in DNA; as such, loss of Stag2 has resulted in a deficiency of homologous recombination-mediated repair of DNA [55]. At double strand DNA breaks, cohesin represses transcription and prevents large-scale genomic rearrangements [56]. In our model system, we did not observe tumor formation in the cohort of mice carrying 3 genetic changes (*EWS-FLI1, p53* null mutation, and *Stag2* loss) without irradiation. It is possible that radiation potentiated the deleterious effect of *Stag2* loss on DNA repair in a *p53* null background and thereby accelerated the accumulation of mutations needed for transformation. More work will certainly be needed to quantify the effect of *Stag2* loss on DNA repair in our model system.

If indeed Stag2 deficiency results in impairment of DNA repair, one might predict that the simultaneous presence of Stag2 and P53 loss would increase the accumulation of mutations and enable tumors to become more aggressive. It is interesting to note that in human Ewing sarcoma, tumors harboring both *STAG2* and *P53* mutations have the worst prognosis and shortest survival [11]. The tumors that developed in this study may have been more aggressive because of irradiation. They were categorized as pleomorphic sarcomas, which is a designation based primarily upon traditional morphologic findings and not specific genetic change. While the literature on mutational changes in pleomorphic sarcomas is sparse, a recent study on human soft tissue sarcomas found only occasional mutations in the cohesin complex, suggesting that *STAG2* mutation is not a common mechanism for the development of pleomorphic sarcomas [57].

While the possibility that *STAG2* is involved in DNA repair, it is important to recall that there may be other aspects of *STAG2* pertinent to its role in transformation, and the function of the *STAG2* gene might not be fully understood at present. STAG2 is expressed broadly in many different cell types, and yet *STAG2* mutation is especially frequent in certain malignancies, including bladder cancer, uterine cancer, and Ewing sarcoma [53]. This hints at the possibility that the contribution of *STAG2* mutation to transformation may be tissue specific and not purely a matter of DNA repair. In a mouse model of leukemia, *Stag2* mutation seems to affect genes involved in hematopoietic stem cell renewal and differentiation [58]. Whether similar mechanisms may be at play in Ewing sarcoma, which is also a malignancy of primitive cells, is interesting to ponder.

One effect that we observed was that inhibition of *Stag2* increased the invasiveness and migration of cells. However, these in vitro properties alone did not predict in vivo tumorigenesis. Loss of *Stag2* alone produced no tumors, and other genetic changes were clearly needed. Some authors have observed that *STAG2* encompasses transcriptional co-activation domains and motifs that may affect cell cycle gene expression [59, 60], but knock-in and knock-out studies found only a slight effect of *STAG2* expression on cellular growth in glioblastoma, human colorectal, and bladder cancer cells [28, 50]. We also did not find an appreciable effect on the growth rate of mesenchymal cells with *Stag2* inhibition. Interestingly, in U2OS cells, the truncating R216 mutation of *STAG2* reduces proliferation but increases invasiveness of cells, which parallels our findings [61]. The co-localization of cohesin-Stag2 to master transcriptional regulatory complexes [62, 63] affords one possible mechanism for *Stag2* loss to affect global processes such as migration and invasion, but this idea will need further testing. Quite possibly, other pathways and systems may be involved. More recent work has shown that loss of *STAG2* increases telomere recombination and postpones replicative senescence in cultured normal human cells [64]. Another interesting observation is that germline mutation or loss of the gene results in mental retardation [65] and craniofacial defects [66]. Together, these observations in aggregate would support the view that *STAG2* has complex pleiotropic effects, which can be radiation-independent, as in the case of migration of cells, but also radiation-sensitive, as in the repair of DNA damage.

The human cohesin complex encompasses 4 main proteins that include SMC1, SMC3, and RAD21 in addition to either STAG1 or STAG2 [12–14]. In our study, we found that the expression of the cohesion complex subunits appeared to be coordinately affected with *Stag2* inhibition. These results are similar to other authors’ findings that *STAG2* mutations decrease the cohesin complex levels and alter its function [27, 28]. In glioblastoma cells, however, no difference in the levels of SMC1, SMC3 and RAD21 was detected upon *STAG2* repression [67]. The discrepancy might point to the importance of cell-specific context in *STAG2* function.

A limitation of the present study is that the analysis was restricted to cells that were *p53* null. Additional cohorts of cells and mice bearing wild-type *p53* would be necessary to determine whether the synergy between EWS-FLI1 and Stag2 occurs in wild-type *p53* cells and whether the individual contributory roles of Stag2 loss and EWS-FLI1 to tumor formation as well as chromosomal aberrations, migration, invasion, and growth in soft agar. In particular, it would be informative to determine whether the effects of Stag2 might be independent of p53, since some human Ewing tumors carry *STAG2* mutation without *P53* mutation. However, we emphasize that the combination of *EWS-FLI1* and *Stag2* loss alone was insufficient to generate tumors in our system. In the current model, irradiation was required, suggesting that other unidentified factors may be critical to tumor development.

## Conclusions

In summary, we show that loss of *Stag2* cooperates with *EWS-FLI1* and *p53* mutation to promote sarcomagenesis in murine MSCs. However, these three genetic changes together are not quite sufficient to produce full transformation of MSCs. Irradiation was necessary for tumors to form, suggesting that additional, as yet unidentified genetic perturbations may play a key role. The mechanism by which *Stag2* inhibition promotes sarcomagenesis is not clear, as it does not seem to affect the proliferation rate or aneuploidy, but it does increase migration and invasiveness. Our data suggests that Stag2 has complex pleiotropic effects on the transformation of EWS-FLI1 bearing cells, which may include both radiation-independent effects as well as radiation-sensitive processes. More work will be needed in the future to address these questions.

## Data Availability

The data used and obtained to support the findings of this study are available from the corresponding author upon reasonable request.

## Abbreviations

ANOVA: analysis of variance
BCA: bicinchoninic acid
CI: confidence interval
CO_2_: carbon dioxide
Ctrl: control
DNA: deoxyribonucleic acid
EDTA: ethylenediaminetetraacetic acid
FACS: fluorescence-activated cell sorting
FBS: fetal bovine serum
GFP: green fluorescent protein
gm: gram
Gy: Gray
MEMα: Minimum Essential Medium alpha
mL: milliliter
MSC: mesenchymal stem cells
MTT: 3-[4,5-dimethylthiazole-2-yl]-2,5-diphenyltetrazolium bromide
NIH: National Institutes of Health
PBS: phosphate buffered saline
PVDF: polyvinylidene difluoride
RIPA: radioimmunoprecipitation assay
RNA: ribonucleic acid
Rplp0: ribosomal protein lateral stalk subunit P0
RT-PCR: reverse transcriptase polymerase chain reaction
SA2: stromal antigen 2
SDS: sodium dodecyl sulfate
shRNA: short hairpin ribonucleic acid
STAG2: stromal antigen 2
TBST: Tris-buffered saline containing 1% Tween 20
WT: wild type
